# When a leader job resource can be ambivalent or even destructive: Independence at work as a double-edged sword

**DOI:** 10.1371/journal.pone.0217482

**Published:** 2019-05-23

**Authors:** Paola Gatti, Michelle C. Bligh, Claudio G. Cortese

**Affiliations:** 1 Department of Psychology, Università degli Studi di Torino, Turin, Italy; 2 Division of Behavioral & Organizational Sciences, Claremont Graduate University, Claremont, CA, United States of America; Radboud University, NETHERLANDS

## Abstract

Independence at work is commonly considered a job resource which fosters motivation and employee well-being. Somewhat paradoxically, it is embedded in a relationship, and employees’ independence also hinges on their leaders’ willingness to grant it. Analyzing this resource as part of the leader-follower relationship can be useful in exploring its beneficial, ambivalent, or detrimental reciprocal effects. We present two Actor-Partner Interdependence Models (APIM) which analyze leaders’ and followers’ independence as antecedents, and work engagement and emotional exhaustion as outcomes. We test our models on 112 pairs of UK workers, finding a significant partner effect between leaders’ independence and followers’ exhaustion. Our findings confirm the utility of a dyadic perspective for investigating leadership and well-being at work, and suggest improvements for leadership training and measures fostering job well-being.

## Introduction

Independence at work is a job resource (viz., an aspect of the job that works as a health protecting factor, see [1, 2]) which is dependent on having interpersonal connections [3]. As such, it is enacted in the framework of a relationship. Since the first and most influential work relationship is, of course, that between a leader and a follower [4], it seems particularly appropriate to investigate autonomy in the leader-follower dyadic relationship to shed light on its “interdependent nature”.

Interdependence, and thus the mutual influence between people in a relationship [5], is a basis for social exchange theory [6], which has been used extensively to study leadership (see the leader-member exchange literature). Interdependence between leaders and followers has been recognized in seminal work on leadership (see e.g., [7–9]), but is much less frequently analyzed [10]. Gooty, Serban, Thomas, Gavin, and Yammarino ([11], p. 1093) point out that “most other domains studying interpersonal relationships and dyads (e.g., family and child psychology) now recognize that ‘relationships’ are best studied via data gathered from both parties to the relationship”. Despite this awareness, the leadership literature tends to “underplay the socially constructed and reciprocal relationship between leaders and followers” ([12], p. 628).

This study’s underlying idea is that leadership is essentially a (mutual) relationship (though with an imbalance in power): in the framework of this relationship, it is thus important to investigate all the dimensions which may be influenced by the leadership dynamic, such as independence at work which has a relational basis by definition. We carry out such an investigation by testing two actor-partner interdependence models (APIMs, which focus on interdependence within interpersonal relationships, see [5, 13]). As Ferrin, Bligh, and Kohles [14] point out, using APIM models may reveal critical interdependencies in how leaders and followers impact one another’s work and life at work. To test our models, we chose two classical outcomes from the job demands-resources model (JD-R model [1]) which is the frame of reference for our study and the model most frequently used to investigate well-being at work in recent years. These outcomes are work engagement and emotional exhaustion [2]. We hypothesize that–in addition to the expected actor effects for both leaders and followers which the JD-R literature has shown (i.e., autonomy increases work engagement and decreases emotional exhaustion, or more classically buffers the effect of certain demands on exhaustion)–there will be partner effects from leaders’ autonomy to followers’ outcomes. Leaders’ independence at work could make exchanges with followers less frequent and followers could thus receive fewer resources, and poor information in particular. In terms of leadership styles, there may be some overlap between leaders’ independence at work and laissez-faire leadership [15], and between the former and other negative leadership styles (e.g., authoritarian leadership). Thus, it is easy to imagine that the relationship between leader independence and follower outcomes is complex, and that a leader’s independence may actually diminish followers’ motivation and increase their emotional exhaustion. Looking for a potential downside of independence at work is also consistent with the well-being literature which, however, deals with one’s own autonomy. Warr’s vitamin model in fact suggested that variables regarded as providing an “opportunity for personal control”–explicitly including autonomy–are healthy at low to moderate levels but become harmful at very high levels [16, 17].

This paper, which could add to the literature on the downsides of resources (see [16]) and on the downsides of leadership dimensions (see e.g., [18], for a shortcoming of transformational leadership), bridges two important gaps in the literature. First, we explore whether a classical job resource like independence at work can be potentially neutral or even harmful from a relational perspective in pairs of leaders and followers. Second, we explore how leader-follower interdependence shapes motivation and well-being at work through the action of an intrinsically relational job resource.

### Independence at work as a job resource in the JD-R model

Independence at work (IaW), or job autonomy, is commonly considered to be conducive to well-being and motivation at work. It is a job resource in the JD-R model [1], the frame of reference for this study, where it is described as a health-protecting factor. Many other models and theories underlying the JD-R emphasize IaW’s importance for well-being and motivation outcomes. These models include the Job Characteristics Model [19], self-determination theory [20], and the demand-control model [21, 22]. It should also be noted that when Hackman and Oldham provide an example of a job characterized by high autonomy, they single out the employee’s leader and the relationship between the two. Specifically, they state ([19], pp. 257–8): “to the extent that a job has high autonomy, the outcomes depend increasingly on the individual’s own efforts, initiatives, and decisions *rather than on the adequacy of instructions from the boss* or on a manual of job procedures” (emphasis added). The authors thus indirectly recognize that IaW is embedded in the leader-follower relationship, but this insight has not yet been analyzed in any depth.

Focusing on the JD-R, the first description of the model mentioned job control as an organizational resource [1], while the later paper on the ‘state of the art’ of the model mentions autonomy as a task resource [2]. Bakker and colleagues ([23], p. 172) defined job autonomy as “on the one hand, independence from other workers while carrying out tasks, and on the other, decision latitude concerning one’s work pace and phases”. This definition clearly separates this construct from others falling under the heading of job control (e.g., participation in decision-making and influence in the organization) and meshes well with the job autonomy scale used in the current study (a later version of that employed, for example, by Schaufeli et al. [24], linking autonomy and work engagement). IaW is also one of the most frequently used job resources in the JD-R model and its impact has been demonstrated on different outcomes: it increases extra-role performance through its relationship with (dis)engagement [25]; it increases organizational commitment and task enjoyment particularly when job demands are high [26]; it increases work engagement [24, 27], and buffers the effect of demands on health impairment outcomes such as emotional exhaustion [23] more than other resources [28]. Considering outcomes, our interest focuses on work engagement and emotional exhaustion, as these are classical outcome variables for the JD-R model. Work engagement is defined as “a positive, fulfilling, work-related state of mind that is characterized by vigor, dedication, and absorption” ([29], p. 74) and is a motivational construct [30]. Emotional exhaustion is the central characteristic of burnout [31] and can be described as a feeling of being emotionally overloaded and worn out [1]. The JD-R model suggests that resources have a direct influence on motivational outcomes while buffering the impact of job demands on health impairment outcomes [2]. Following the literature on the JD-R model and earlier approaches which discuss autonomy as linked to motivation and well-being at work from different perspectives, we hypothesize that:

Hypothesis 1. Respondents’ IaW has a positive relationship with their own work engagement and a negative relationship with their own emotional exhaustion.

Hypothesis 2. IaW has a stronger link with work engagement than with emotional exhaustion, because of the more direct relationship with the former.

These two hypotheses have been tested in previous studies (see e.g., [27] and the meta-analysis by Halbesleben [32] for the relation between IaW and work engagement; see [28] for the relation between IaW and emotional exhaustion), but testing them again with the APIM models can provide further confirmation from a different perspective. In addition, the JD-R model does not explore the influence of interdependence at work on the operation of resources (and demands), even though some of them are explicitly dependent on relationships (e.g., supervisor support, role clarity and role conflict, as well as job autonomy) and even though it recognizes the importance of leadership as a potential job resource [33]. Moreover, Bakker and Demerouti [33] emphasize that future applications of the JD-R model “should take the multilevel nature of data into consideration” (p. 278), an idea which is consistent with our use of the dyadic approach.

### Toward a relational (leader-follower) approach for independence at work

The leadership literature, like the studies which adopt the JD-R framework, also emphasizes the positive aspects of autonomy. IaW is sometimes described as a resource for developing a good relationship between a leader and a follower (see the leader-member exchange literature, which argues that “negotiating latitude” granted by the supervisor at the outset is predictive of the subsequent quality of the relationship [8]), and sometimes as an outcome of good leadership (see transformational leadership literature which maintains that this leadership style “fosters autonomy and challenging work”, [34], p. 10). By contrast with the literature on motivation and well-being at work, leadership studies offer more grounds for considering autonomy as a resource enacted in the framework of a relationship.

IaW’s relational value is thrown into even sharper focus by the dialectical approach to personal relationships [3], which has recently been adopted to understand one’s relationship with the supervisor [35]. Most dialectical theories center on functionally defined opposites, where the elements of the contradiction are each functionally unique, but negate each other in some way. Autonomy can thus be considered as opposite to connectedness, and exists in the context of connection [35]: having autonomy is dependent on having interpersonal connections [3]. In an organization, there can be little doubt that the primary interpersonal connection, the most important of all relationships, is that between leader and follower: a relationship of mutual influence [36] which offers a lens through which the follower can view the entire work experience [4]. It is thus critical to study IaW in the framework of the leader-follower relationship, using a relational approach to recognize the inherent interdependencies characterizing this relationship. To date, most IaW investigations have relied on samples of followers, or to be more precise on samples where the respondents’ position in the hierarchy was not taken into consideration and people thus answered the questions about autonomy from the standpoint of their role as supervisees. A few studies have used hierarchical position as a variable in some way (e.g., Mauno, Mäkikangas, and Kinnunen [37] asked respondents whether or not they had a managerial position and use that variable as a control). Supervisors, as expected, have higher autonomy levels than supervisees [38], and autonomy is also influential as a job resource for them (e.g., [39]). All in all, there are far fewer investigations of leaders or of leader-follower relationships with respect to independence.

Nevertheless, dyadic investigations are highly topical, not only for leadership scholars, but also for job design scholars for whom a relational approach is important, given the recent increase in interdependence at work [40]. Actor-partner interdependence models (APIM, [5]) analyze interdependence between pairs, and are thus particularly suitable for the present study. So far, to our knowledge, no studies investigate job autonomy using the APIM’s dyadic approach, despite the fact that the need for autonomy has been investigated with this analysis technique, e.g., in romantic relationships [41]. We present the findings from two APIM models which analyze IaW as an antecedent and work engagement or emotional exhaustion as outcomes. While we are aware of the shortcomings of investigating the impact of a resource on emotional exhaustion from the JD-R standpoint (see [33] and the different paths described for resources and demands towards the outcomes), here our main focus is on interdependence and partner effects. Specifically, we argue that some resources (and potentially some demands) could operate quite differently within a dyad than at the individual level that predominated in the development of the JD-R model, and that, more in general, it is highly relevant to study job resources (and demands) that have a relational basis, such as IaW, in the framework of the leader-follower relationship.

#### Ambivalences of independence at work in the leader-follower relationship

Independence at work seen in the relationship between a leader and a follower shows two main ambivalences. The first is linked to the fact that the relationship between leaders and followers entails a difference in power, and followers are by definition in subordinate roles with less power [42]. As a result, the relational resource of autonomy is likely to be particularly salient and critical for followers, who think of their autonomy as something to be negotiated–since they have to “learn to participate effectively in the [leadership] relationship despite this [power] imbalance” ([43], p. 18). Followers’ IaW, as it is a resource that has to be “earned” from their standpoint, could thus be a stronger predictor of followers’ motivation and well-being outcomes than leaders’ IaW. We hypothesize:

Hypothesis 3. The relationship between followers’ IaW and the followers’ outcomes is stronger than the relationship between leaders’ IaW and their own outcomes.

The second ambivalence emerges when we focus on leaders’ IaW. This is not the first situation where otherwise positive aspects of leadership have been found to be harmful or dangerous. For instance, LMX has shown functional ambivalences and curvilinear relationships with followers’ outcomes, in line with Warr’s theorization (see for instance Hochwarter and Byrne [44], who found a curvilinear relationship between LMX and job tension, where very positive relationships are associated with a renewed increase in tension).

Leader IaW’s ambivalent status in the leadership literature can likewise be linked to LMX theory. LMX is built on social exchange theory [45, 46] which argues that any social exchange involves a series of interactions which generate obligations [46]. These interactions are seen as interdependent and contingent on someone else’s actions [45] and can potentially generate high-quality relationships [6]. The exchange process has certain “rules” that should be respected, the first is that of reciprocity. Gouldner [47] described three types of reciprocity, one being reciprocity “as a transactional pattern of interdependent exchanges” [6]. Interdependence is a defining characteristic of social exchange and is a condition which lies between the two extremes of dependence and independence. These last two conditions do not imply a social exchange [6]. Here we posit the ambivalence of leaders’ IaW and its potential harmful effect on followers. Highly independent leaders may not nurture the potential social exchange with their followers that can lead to a high quality relationship. The exchange between the leader and the follower could thus be weak, and the concrete occasions for meeting and sharing resources could be infrequent.

From another standpoint, that of leadership styles, IaW’s ambivalent status is also evident if we consider leaders’ independence together with laissez-faire leadership–a negative style of leadership which is marked by an absence of leadership and the avoidance of intervention [15]. Laissez-faire leadership can be interpreted as a form of excessive independence enacted by the leader. According to some authors, for instance, laissez-faire leaders show a lack of response to supervisees’ needs and performance [48, 49] and do not meet supervisees’ legitimate expectations [50]. Though the literature on job autonomy, as well as the items in the IaW scale used for this investigation, clarify that independence regards one’s own work and activities, leaders’ activities entail making decisions and sharing information which influence their supervisees’ life on the job. Leaders who think of themselves as highly independent may not involve their supervisees in such decisions or share important information with them, and thus fail to meet their followers’ legitimate expectations of being involved in the work process. In addition, laissez-faire leadership does not appear to be motivated and intentional [48]. Since it is not a deliberate harmful behavior, such leaders may think of themselves as independent instead of avoidant. There is thus some evidence that followers could interpret leaders’ IaW as ambivalent, or even negative.

Though we have singled out the laissez-faire leadership style as the clearest example of leader IaW’s ambivalence, there are other negative leadership styles where leaders could describe themselves as highly independent at work. One of these styles is authoritarian or autocratic leadership, where the leader determines all decisions in the organization [51]. Here, we do not mean to affirm that leaders who describe themselves as highly independent are necessarily destructive leaders or want to be destructive; specifically, this is why we concentrate on the possible link between leaders’ IaW and laissez-faire leadership which, as Schyns and Schilling argue [52], is not strictly speaking a destructive style. At the same time, however, followers could perceive such independent leader behavior as a sign of bad leadership. This is easy to understand, considering the emphasis that the literature on positive leadership styles puts on the importance of informing followers and interacting with them (see for instance the empowering leadership literature, e.g., [53]) as well as the impact of developing a leadership relationship with followers instead of a more detached and authority-based supervision exchange (see the LMX literature, e.g., [8]). The underlying idea is that leaders who describe themselves as highly independent may show, or be perceived to show, some of the behaviors of laissez-faire or even authoritarian leaders, and that their independence can thus have an ambivalent or even negative effect on their followers. Given the difference in power, the potential for leaders’ high IaW to weaken the exchange with the follower, and also the potential for leaders’ high IaW to be perceived by their followers as a part of a negative leadership style, we might also expect that leader’s IaW will diminish followers’ motivation and increase health impairment. We thus hypothesize that:

Hypothesis 4. Leaders’ IaW decreases followers’ work engagement and increases followers’ emotional exhaustion.

## Method

### Sample and procedure

We examined these hypotheses in a 112-dyad sample of UK workers (supervisees and their direct supervisors) from five subsamples in different sectors. Specifically, our sample was drawn from four organizations and a fifth subgroup of 25 pairs of leaders and followers working in several SMEs in UK (this subgroup initially consisted of a larger number of pairs, but only those whose members’ leader/follower roles and correct matchup could be verified were retained). Participating organizations opted for different levels of involvement: two accepted having the questionnaire administered to pairs from their entire workforce (and thus provided their complete organization chart to the researchers, who then chose pairs randomly), while two chose a number of pairs following our instructions, and gave a list to the researchers who then selected the pairs for the sample. Data were collected from November 2014 to October 2015.

Most questionnaires were administered online (via the Qualtrics platform), as only one company chose a paper-and-pencil administration. Potential participants received an email invitation with a link to the online questionnaire or the logistics details for the paper-and-pencil administration. The invitation and an information sheet given to participants immediately before the administration or presented as the first page of the online questionnaire specified the project’s ultimate goal, the instructions about the paired partner to think about when answering some of the questions, the fact that participation in the project was absolutely voluntary, all answers were completely confidential (with no obligation to respond), and that by filling in the questionnaire the respondent was consenting to take part in the study. Participants knew exactly who they were paired with for the project as well as their own role (as a leader or a follower) in it: a separate email provided each respondent with the name of his/her paired partner and the alphanumeric code whereby the person and the pair could be identified without using their names when completing the questionnaire. The study thus complied with the Helsinki Declaration [54] and was approved by the Ethics Committees of the researchers’ university and the funding organization.

Lastly, we are aware that this operationalization of the leader-follower relationship raises certain questions, since supervisees are not always followers and direct supervisors are not always leaders [55]. Nevertheless, we chose this approach, which is followed by other important studies of these topics (e.g., [56]), because our focus is on interdependence. In other words, some supervisees might not regard their direct supervisor as their leader, but the type of IaW which could more directly impact their well-being is the autonomy that a person with a concrete relationship with the supervisee enacts, and the autonomy of a person who can make decisions capable of influencing the supervisee’s everyday life at work.

Our final sample thus consisted of 112 respondents who filled in the follower questionnaire, and 112 respondents who filled in the leader questionnaire. From the socio-demographic standpoint, the two subsamples are fairly similar, showing only slight differences: the supervisor subsample includes a higher proportion of males and older people, with a somewhat higher education level and somewhat longer tenure in the organization. The 112 dyads had been working together for around 27 months on average (*SD* = 31.31), and 72.3% worked in the same office or space. Table 1 details the socio-demographic characteristics of the sample.

**Table 1:** Socio-demographic characteristics of two subsamples of leaders and followers.

Socio-demographic variables	Type of answer /	Followers	Leaders
	Type of analysis		
	(if average value)		
Sex	Female	67.3%	63.4%
	Male	32.7%	36.6%
Age	*M*	39.03 years	41.67 years
	*SD*	11.20	9.64
Educational Level	GCSE	19.8%	13.5%
	A levels	21.6%	13.5%
	Graduate degree	42.4%	47.8%
	Postgraduate degree	16.2%	25.2%
Work schedule	Full-time	89.0%	93.6%
	Part-time	11.0%	6.4%
Tenure in the organization	*M*	8.95 years	11.01 years
	*SD*	7.03	7.29

### Measures

The questionnaire was quite long and divided into 4 sub-sections, viz.: 1) socio-demographic characteristics, 2) personal and job characteristics, 3) leadership and followership, and 4) attitudes and behaviors at work. For this study, we used only the scales of IaW (section 2), of work engagement and emotional exhaustion (section 4).

*Independence at Work* (IaW) was measured with the four items developed by van Veldhoven, Prins, van der Laken and Dijkstra [57] for the Questionnaire on the Experience and Evaluation of Work (QEEW2.0), which were on a 4-point Likert scale (1 = never, 4 = always). A sample item is: “Do you have freedom in carrying out your work activities?”. Cronbach’s alpha for this scale was .74 for followers and .82 for leaders.

*Work engagement* (WE) was measured with the 9 items developed by Schaufeli, Bakker, and Salanova [58], which were on a 7-point Likert scale (0 = never, 6 = always). A sample item is: “At my work, I feel bursting with energy”. Cronbach’s alpha for this scale was .91 for followers and .89 for leaders.

*Emotional exhaustion* (EE) was measured with the 8 items developed by Demerouti, Mostert, and Bakker [59] as part of the Oldenburg Burnout Inventory (OLBI), which were on a 4-point Likert scale (1 = strongly disagree, 4 = strongly agree). A sample item is: “After my work, I usually feel worn out and weary”. Cronbach’s alpha for this scale was .83 for followers and .80 for leaders.

### Data analysis

We conducted descriptive statistics on all variables in our study to describe the sample and compared mean values of our antecedent and two outcomes in the two subsamples of leaders and followers. Independent-sample t-tests on the pairwise data matrix were used to determine differences in IaW, work engagement and emotional exhaustion between respondents in the two subsamples (see Table 2 for descriptives and t-tests). On the three scales, the measure of Cronbach’s alpha was used to test for homogeneity and internal consistency [60]. Correlations between the predictor and the outcomes in leader-follower dyads and in each subsample are given in Table 3 together with the partial Pearson correlations to test for nonindependence [61]. Nonindependence means that the two members of a dyad share something in common, which makes their scores on a scale more similar to or more different from one another than the two scores of two people who are not paired [13]. This analysis can be considered as a test of whether data should be analyzed dyadically: if we ignore nonindependence in the dependent variable and analyze data at the level of the individual, we will obtain biased parameter estimates [61].

**Table 2:** Mean values on the study variables and t-tests for two subsamples.

Dimension	Followers’ *M* (*SD*)	Leaders’ *M* (*SD*)	t-test	*p*
Independence at Work	3.19 (0.53)	3.34 (0.49)	2.20	< .05
Work Engagement	4.23 (1.05)	4.42 (0.91)	1.14	*ns*
Emotional Exhaustion	2.20 (0.61)	2.25 (0.56)	0.62	*ns*

**Table 3:** Individual- and dyad-level correlation coefficients between the investigated variables.

Variables	1.	2.	3.	4.	5.	6.
1. IaW (F)	-					
2. IaW (L)	.29**	-				
3. WE (F)	.28**	-.02	-			
4. WE (L)	.05	.26**	**.04**	-		
5. EE (F)	-.15	.20*	-.43***	.17	-	
6. EE (L)	.11	-.19*	.10	-.38***	**-.15**	-

*p < .05

**p < .01

***p < .001

*Note*. (F) = followers; (L) = leaders; IaW = Independence at Work; WE = Work Engagement; EE = Emotional Exhaustion. In bold, partial Pearson correlations to test for nonindependence.

**Fig 1:**
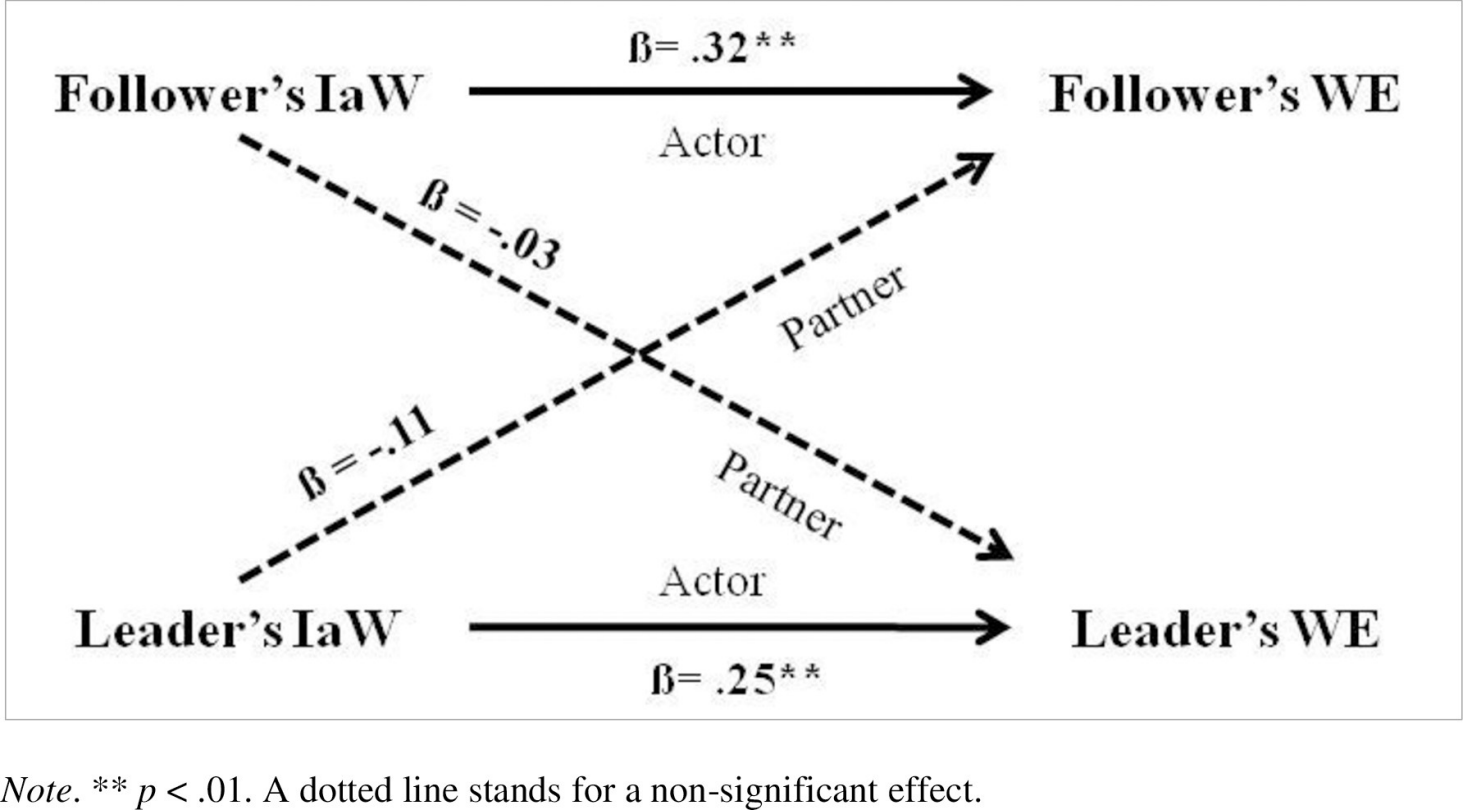
APIM with distinguishable dyads: Independence at Work (IaW) on Work Engagement (WE).

**Fig 2:**
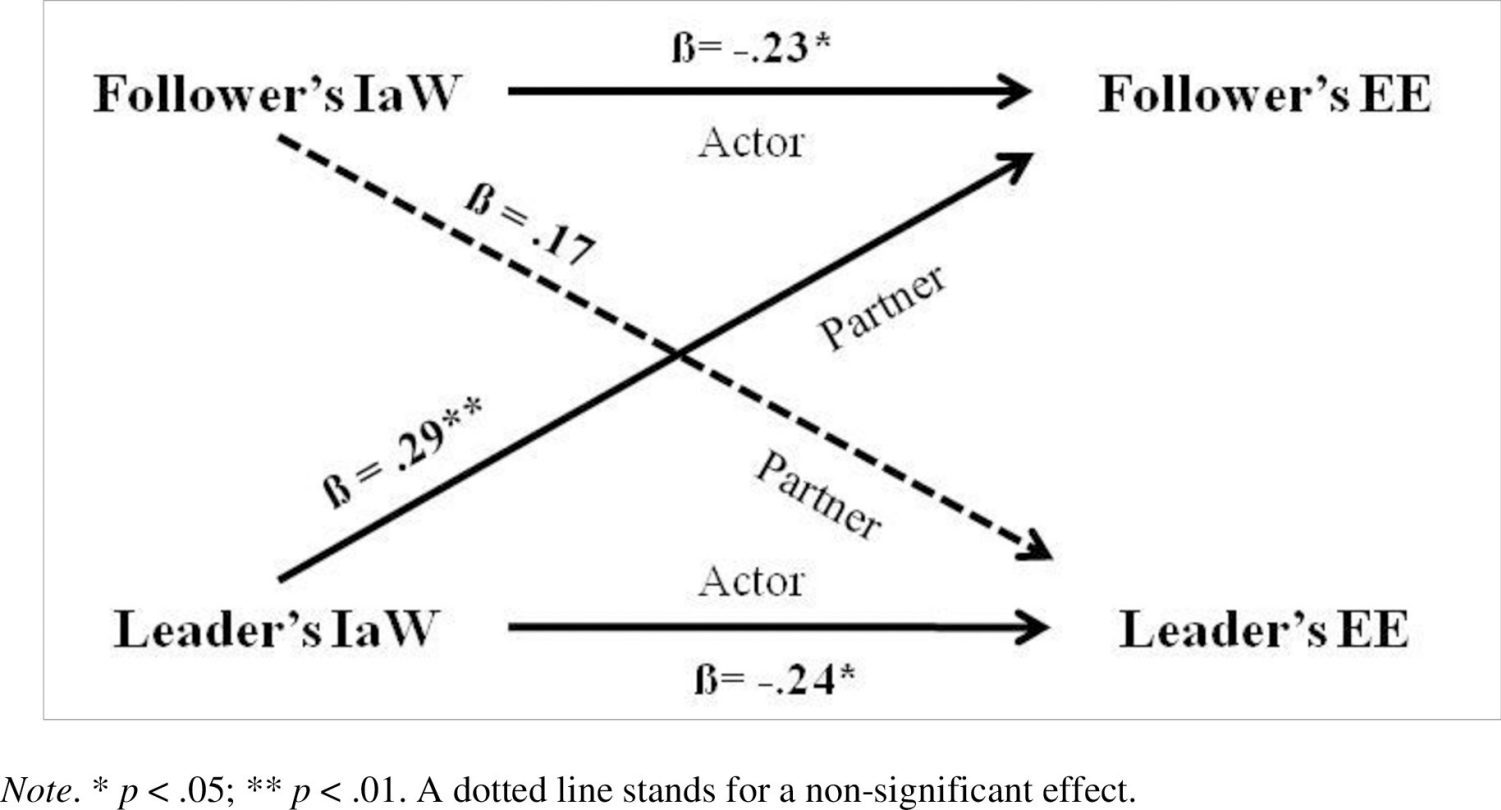
APIM with distinguishable dyads: Independence at Work (IaW) on Emotional Exhaustion (EE).

To determine the impact of leaders’ and followers’ IaW on their own work engagement or their own emotional exhaustion as well as on their partner’s outcomes, we applied the Actor–Partner Interdependence Model (APIM; [5, 13]). APIM regression is used to determine how outcomes are influenced by both members of the dyad and the interdependence between the two members of the pair. For this study, the actor effect was the impact of a person’s IaW on his or her own work engagement or emotional exhaustion. The partner effect was the impact of each person’s IaW on his or her partner’s work engagement or emotional exhaustion. The two models were estimated using SPSS 22 software and thus the multilevel modeling (MLM) method for analysis. To test the models, we centered the independent variable of IaW (both the actor and partner values) and tested an interaction model and a two-intercept approach model, first using these scores. We then re-tested the two models using z-scored variables (i.e., standardized coefficients); these values are given in Figs 1 and 2. Lastly, we determined the variance explained by each model by calculating a Pseudo R-square [5]. At least in part, data presentation is based on that suggested by Chung, Moser, Lennie, and Raynes [62] in their paper using APIM models.

In addition, in order to investigate whether followers’ actor effects are stronger than leaders’ actor effects as stated in Hp. 2, we performed our APIMs again using the app described by Stas, Kenny, Mayer, and Loeys [63]. This also allowed us to compare partner effects in each model (to answer Hp. 3). The app does so by estimating APIM using structural equation models (SEM) and providing a *p* value of the test to measure whether two actor (or two partner) effects in a model are equal or not. Lastly, this app also performs a test for distinguishability which will be discussed below.

## Results

Levels of IaW differed significantly between leaders and followers, while levels of work engagement and emotional exhaustion did not (see Table 2). Specifically, leaders perceived higher independence at work than followers (leaders’ mean = 3.34, followers’ mean = 3.19; t = 2.20, p < .05), but in both subsamples the level of IaW was quite high.

Followers’ IaW is significantly correlated with followers’ work engagement (*r* = .28, p < .01) but not with their emotional exhaustion or the outcome variables as measured on leaders, while leaders’ IaW is correlated with both leaders’ (r = -.19, p < .05) and followers’ (r = .20, p < .05) emotional exhaustion and with their own work engagement (r = .26, p < .01), but not with that of followers. The correlation between leaders’ IaW and followers’ emotional exhaustion is thus positive. Table 3 shows partial Pearson correlations (in bold) to test for nonindependence. The correlations between leaders’ and followers’ WE and leaders’ and followers’ EE are not significant, though in the latter case the correlation is close to being significant. Using APIMs, though not strictly necessary, is thus of particular interest.

Two APIM models were tested to examine our hypotheses. In one, IaW was regressed on work engagement, while in the second IaW was regressed on emotional exhaustion. For work engagement, IaW showed both leaders’ and followers’ actor effects, while there were no significant partner effects (see Table 4 and Fig 1). Leaders’ and followers’ with higher IaW had higher engagement in their work but their own IaW did not have an impact on the partner. For emotional exhaustion, IaW showed both leaders’ and followers’ actor effects, while there was also a significant partner effect from leaders’ IaW to followers’ emotional exhaustion (see Table 4 and Fig 2). Leaders and followers with higher IaW had lower exhaustion, but the higher the leaders’ IaW, the higher the followers’ emotional exhaustion.

**Table 4:** Actor and Partner relationship of IaW and WE and of IaW and EE.

	Leaders			Followers		
Effect	β	t	p	β	t	P
**On WE**						
Actor	.25	2.70	.008	.32	3.18	.002
Partner	-.03	-0.32	.749	-.11	-1.07	.288
**On EE**						
Actor	-.24	-2.46	.015	-.23	-2.38	.019
Partner	.17	1.87	.064	.29	2.74	.007

*Note*. IaW = Independence at Work; WE = Work Engagement; EE = Emotional Exhaustion.

Lastly, we calculated the Pseudo R-square for the first model on work engagement, which is significant and equal to .16 [Pseudo R^2^ (Leaders) = .07 and Pseudo R^2^ (Followers) = .09; χ^2^ (2) = 17.697, p < .001], and the Pseudo R-square for the second model on emotional exhaustion, which is again significant and equal to .16 [Pseudo R^2^ (L) = .07 and Pseudo R^2^ (F) = .09, χ^2^ (2) = 15.734, p < .001].

Hp. 1 is thus confirmed, since actors’ IaW has a positive impact on their work engagement and a negative impact on their emotional exhaustion. Regarding Hp. 2, the coefficient of IaW is higher in modulus on WE than on EE for followers but is very similar for leaders, so there seems to be a stronger link between IaW and work engagement than between IaW and emotional exhaustion for followers than for leaders. In partial contradiction with this, however, the two Pseudo R-squares of the models are equal and thus the variance explained by the models is equal, and not higher when the outcome is work engagement as we would have expected. Unfortunately, we cannot test for the statistically significant difference between the effects on our two outcomes by using APIMs, as this would require another analysis (i.e., multilevel SEM) and is thus out of the scope of this study. Regarding Hp. 3, as stated above, followers’ IaW has a higher coefficient on work engagement than leaders’ IaW, but the difference on emotional exhaustion is minimal; however, followers’ EE shows a significant partner effect from leaders. In sum, IaW seems to be more important for followers’ WE than for leaders’ WE, but the same cannot be said for EE. Using the app by Stas and colleagues [63], we checked the difference between the two actor effects in each model, finding that it was not significant, even in the case of WE where the difference in module is higher and, as expected, the effect for followers is stronger than for leaders.

Hp. 4 is partially confirmed, since leaders’ IaW increases followers’ emotional exhaustion but does not decrease followers’ work engagement. Although we did not make a hypothesis for partner effects from followers to leaders, the partner-effect from followers’ IaW to leaders’ EE deserves a mention, since it is close to being significant (see Fig 1), and again using the app, this shows that the difference between the two partner effects on EE is not significant, even though the partner effect from leaders to followers is significant and that from followers to leaders is not. The overall partner effect from IaW to EE would be significant even if we consider our dyads as indistinguishable. This would deserve attention in future research. Strictly speaking, and using the test for distinguishability performed by the app, this shows that from a statistical standpoint our pairs cannot be distinguished on the basis of their role as followers or leaders. However, we chose to comment on APIMs on distinguishable dyads because of the research design which underlies our data collection (where participants were informed of the role they would have in the project, i.e., whether they would be categorized as leaders or followers), and because we were interested in emphasizing each specific characteristic of our two subsamples and thus all the trends in our data. In addition, our approach is confirmed by some scholars who are experts on the method and wrote that: “distinguishability should be seen as a conceptual matter that needs to be resolved a priori on the basis of whether the members can be clearly distinguished from one another using a theoretically defendable defining characteristic (e.g., gender, *senior vs*. *junior in dyadic work unit* [emphasis added], backcourt vs. net players in a badminton double team).” ([64], p. 80).

## Discussion and implications

The findings from the two APIM models confirm that there are significant actor effects in both models, showing that IaW increases work engagement and decreases emotional exhaustion for both leaders and followers, and bearing out the role of IaW as an important job resource at an individual level. These effects are slightly different (but not in a statistically significant way): followers’ actor effect on WE has the highest beta coefficient, while leaders’ actor effect is lower, as followers’ actor effect on EE is lower and almost identical to leaders’. This is in line with some JD-R assumptions and a number of considerations voiced in the leadership literature. The innovative finding is the partner effect from leaders’ IaW to followers’ EE: the higher leaders’ IaW, the higher followers’ EE. The study thus sheds light on a downside of the IaW resource when considered in a leader-follower relationship. Just as laissez-faire leadership is linked to followers’ stress [49, 50], this study found that leaders’ IaW is linked to followers’ EE. The fact that there is no significant partner effect from leaders’ IaW to followers’ WE was not expected, but the relationship between the two variables is in the anticipated direction. The stronger partner effect with EE can be explained by the nature of this outcome which is a component of the intrinsically relational syndrome of burnout [65]. It may be that leaders’ high IaW can (paradoxically) burden their followers since it is linked to a weaker exchange between the leader and the follower and to less frequent contacts, resulting in a low level of information exchange between the partners in the relationship. This could be a stressful downside of leaders’ IaW for followers, whose (uninformed) life on the job could cause them greater anxiety. We focused here on the partner effect from leaders to followers, but it should be pointed out that if we consider the model on indistinguishable dyads we would still have a significant partner effect. It would thus be very important to investigate what makes IaW an emotionally demanding characteristic in the leader-follower relationships in general. It might also be rewarding to focus on specific kinds of followers (e.g., those whom their leaders consider to be the best coworkers or those closest to them) to see if this partner effect becomes fully significant while treating dyads as distinguishable.

This finding indicates an important exception to IaW’s operation as a job resource for motivation and well-being at work. Though IaW continues to act as an important resource, and can be very positive for the individual who experiences this condition, it can also have a negative influence on followers when enacted by their leaders and more in general a negative influence in the framework of leader-follower relationships. We might conclude that IaW is an “egotistic job resource”, and in a time when interdependence at work has increased [40] this is a point that merits attention. We could also conclude that it is a job resource with a negative halo effect, which can act as a double-edged sword in a leader-follower relationship. Here we do not suggest that job autonomy should be redefined or in any way limited, but that this important caveat must be borne in mind when discussing it. As a leader, you should grant autonomy to your followers (as the literature on LMX and constructive leadership styles argues) and you should pay attention to the complex role and complex effects of your autonomy (as the literature on leader ethical decision-making–see [66]–suggests), considering the impact it can have on your followers’ life on the job.

Lastly, the theoretical implications of this study center on the value of a dyadic perspective in investigating leadership. This is a point which has already received attention (see for instance [11]), but we would add that it is important to adopt this dyadic perspective by pairing leaders and followers when investigating well-being at work and, more specifically, other JD-R model job resources (as well as some job demands). Two resources which have benefited from APIM studies are trust in management and cooperation [14], but role clarity and participation in decision making (both mentioned by Schaufeli and Taris, [67], in their description of the JD-R model) could also benefit from this kind of investigation.

### Practical implications

More engaged employees are known to show higher job performance [68], and lower turnover intentions [32], while burnout, and exhaustion in particular, leads for instance to higher turnover intentions [69]. Likewise, several well-established theories and models recognize that job autonomy leads to many advantages and positive results. Prior to this study, however, there had been no evidence of the potentially negative impact of leaders’ IaW on followers’ burnout.

All of these relationships emphasize the practical value of this study. Specifically, several practical implications follow from the negative role of leaders’ IaW on followers’ emotional exhaustion. Workers could benefit from training and leadership development programs that address this ambivalent role of job autonomy–which seems to be highly positive when granted by a leader to a follower (e.g., [8, 34]), but not when it reaches very high levels (cf. its curvilinear relationship on well-being outcomes [70]), or when it is enacted by a leader who, in a certain sense, forgets how autonomy is embedded in connectedness [3]. As discussed in the literature on negative leadership styles, some of these styles are voluntarily harmful while others are not [52], so in many cases leaders could simply be unaware of the risks that their IaW entails, while their followers could interpret that independence as part of a destructive leadership behavior. Shedding some light on this downside could thus help improve employees’ well-being at work. In addition, training in good communication–a strong predictor of leader performance [71]–could be important in order to contain this risk and make leaders’ IaW less harmful for followers. A link between communication with supervisors and burnout was established in a pioneering work on the latter topic [65], a finding which our study to some extent reinforces.

### Limitations and future research

Some of the study’s limitations are methodological, while others involve its content. Regarding methodology, we used cross-sectional data and a self-report questionnaire, choices which lead to risks of common method bias and self-report bias respectively. Cross-sectional data, in addition, cannot be used for causal assumptions, but it is also true that the direction of the relationships we tested has been confirmed by the literature (see [27, 32] for the link between IaW and work engagement; see [28] for the link between IaW and emotional exhaustion). In connection with self-report bias, the data can also be affected by an intergroup bias since the two groups of respondents are categorized [72] as leaders and followers. The negative connotations of followership (e.g., [73]) or the heroic view of leadership (see the romance of leadership [74]) could have influenced both subgroups’ answers to some extent. It would thus be interesting to repeat the study using leader-follower pairs whose members are unaware they have been paired for the investigation. Another limitation linked to the methodology is that APIMs cannot be used to test for the statistically significant difference between the effects on two or more outcomes, as this would require a more complex analysis (i.e., multilevel SEM). A final methodological limitation is that the sample is quite heterogeneous and consists of leader and follower pairs from different organizational contexts. It is also true, though, that our main focus was on that kind of IaW that either must be negotiated with the direct supervisor or is enacted in a way that can influence one’s own supervisees, and all the pairs in the sample share this basic characteristic and thus allow us to investigate the topic of interest.

As for the study’s content, one limitation is that we had no measures of leadership styles which permitted a better interpretation of our findings and especially of the partner effect we found. Since our ideas of the potential overlaps between leaders’ IaW and laissez-faire leadership or authoritarian/autocratic leadership could not be investigated further, it would be interesting to replicate the study with some of these additional measures. Lastly, our models did not consider potential moderators: it could be profitable–*cf*. our remarks on good communication–to use followers’ perceived quality of supervisor communication as a moderator, or the frequency of communication, as was used in a dyadic study on work engagement by Bakker and Xanthopoulou [75].

Other future developments, could include: 1) analyzing APIM models over time to see how the link between job autonomy and emotional exhaustion develops longitudinally; since leader-follower relationships begin quickly [76], this would involve considering new leader-follower relationships and their development in the first year or so of work together; 2) investigating IaW’s impact on other well-being outcomes, such as positive and negative emotions at work, job satisfaction or psychological symptoms, to determine whether the same kind of partner effect we found is replicated on other variables making up the multi-faceted construct of well-being; 3) replicating the model using scales which measure job autonomy as a multi-faceted construct (e.g., the scale by Breaugh [77]) or measure linked constructs such as participation in decision making which are part of the more general resource of control, to articulate the partner effects occurring for this resource; 4) trying to clarify, as part of this effort to explain the partner effects of independence at work, whether there is a threshold for leaders’ IaW after which its impact on followers’ outcomes becomes harmful. It would thus be interesting to investigate if there is a curvilinear relationship between these two variables (leaders’ IaW and followers’ well-being outcomes) or, as mentioned above, if there are moderators which can make the impact of leaders’ IaW on followers less harmful; 5) testing APIM models on leaders and followers using other job resources (and job demands), to see if the dyadic perspective identifies trends in partner effects which could suggest additions to the JD-R model.

Again in connection with partner effects, future work should also focus on investigating potential trends in the opposite direction to that discussed here, thus analyzing partner effects from followers’ antecedents to leaders’ outcomes. This could shed new light on followership. This recommendation springs to some extent from the result of one of the APIMs presented here where followers’ IaW increases leaders’ exhaustion in a way which is close to significant. In what circumstances could followers’ impact on their leaders become significant? This question might be the guideline for future developments that are based on this study.

Overall, we emphasize that some job resources (as well as some job demands) are fundamentally embedded in the context of the leader-follower relationship, and are thus explicitly negotiated and experienced from positions of more or less power. In the absence of connection and communication, a leaders’ increased independence at work may be perceived as neglect or even abuse by followers. Alternately, IaW may allow both leaders and followers the choice and empowerment to sustain positive outcomes and performance. Increasingly, more and more jobs would benefit from carefully balancing resources to provide both autonomy and choice as well as connection and collaboration. Analyzing IaW in the framework of the leader-follower relationship can thus be useful in understanding how we can more effectively design jobs and develop leaders capable of negotiating this dynamic.

## Supporting information

S1 Data Pairwise data matrix (SPSS). Data matrix with all information for replicating the study.
(SAV)

## References

[1] Demerouti E, Bakker AB, Nachreiner F, Schaufeli WB. The Job Demands–Resources model of burnout. J Appl Psychol. 2001; 86(3): 499–512. 10.1037/0021-9010 86 3 499 11419809

[2] Bakker AB, Demerouti E. The job demands–resources model: State of the art. J Manage Psychol. 2007; 22(3): 309–328. 10.1108/02683940710733115

[3] Baxter LA, Montgomery BM. Relating: Dialogues and dialectics New York: Guilford Press; 1996.

[4] Gerstner CR, Day DV. Meta-analytic review of leader-member exchange theory: Correlates and construct ideas. J Appl Psychol. 1997; 82: 827–844. 10.1037/0021-9010.82.6.827

[5] Kenny DA, Kashy DA, Cook WL. Dyadic Data Analysis. New York: Guilford Press; 2006.

[6] Cropanzano R, Mitchell MS. Social exchange theory: An interdisciplinary review. J Manag. 2005; 31(6): 874–900. 10.1177/0149206305279602

[7] Burns JM. Leadership. New York: Harper and Row Publishers; 1978.

[8] Dansereau F, Graen G, Haga WJ. A vertical dyad linkage approach to leadership within formal organizations: A longitudinal investigation of the role making process. Organ Behav Hum Perform. 1975; 13(1): 46–78. 10.1016/0030-5073(75)90005-7

[9] Herold DM. Two-way influence processes in leader-follower dyads. Acad Manage J. 1977; 20(2): 224–237. 10.5465/255396

[10] Nahrgang JD, Morgeson FP, Ilies R. The development of leader–member exchanges: Exploring how personality and performance influence leader and member relationships over time. Organ Behav Hum Decis Process. 2009; 108: 256–266. 10.1016/j.obhdp.2008.09.002

[11] Gooty J, Serban A, Thomas JS, Gavin MB, Yammarino FJ. Use and misuse of levels of analysis in leadership research: An illustrative review of leader–member exchange. Leadersh Q. 2012; 23(6): 1080–1103. 10.1016/j.leaqua.2012.10.002

[12] DeRue DS, Ashford SJ. Who will lead and who will follow? A social process of leadership identity construction in organizations. Acad Manage Rev. 2010; 35(4): 627–647.

[13] Cook WL, Kenny DA. The Actor–Partner Interdependence Model: A model of bidirectional effects in developmental studies. Int J Behav Dev. 2005; 29(2): 101–109. 10.1080/01650250444000405

[14] Ferrin DL, Bligh MC, Kohles JC. It takes two to tango: An interdependence analysis of the spiraling of perceived trustworthiness and cooperation in interpersonal and intergroup relationships. Organ Behav Hum Decis Process. 2008; 107(2): 161–178. 10.1016/j.obhdp.2008.02.012

[15] Bass BM, Avolio BJ. Improving Organizational Effectiveness through Transformational Leadership. Thousand Oaks, CA: Sage; 1994.

[16] Warr P. Work, Unemployment, and Mental Health New York: Oxford University Press; 1987.

[17] Warr P. Searching for happiness at work. Psychologist. 2007; 20(12): 726–729.

[18] Anderson MH, Sun PYT. The downside of transformational leadership when encouraging followers to network. Leadersh Q. 2015; 26: 790–801. 10.4236/jssm.2016.93033

[19] Hackman JR, Oldham GR. Motivation through the design of work: Test of a theory. OBHP. 1976; 16: 250–279. 10.1016/0030-5073(76)90016-7

[20] Deci EL, Ryan RM. Intrinsic motivation and self–determination in human behavior. New York, NY: Plenum; 1985.

[21] Karasek RA. Job demands, job decision latitude, and mental strain: Implications for job design. Admin Sci Quart. 1979; 24: 285–308. 10.2307/2392498

[22] Karasek RA. Demand/Control Model: A social, emotional, and physiological approach to stress risk and active behaviour development In: Stellman JM, editor. Encyclopaedia of Occupational Health & Safety. Geneva: ILO; 1998 pp. 34.6–34.14.

[23] Bakker AB, Demerouti E, Euwema MC. Job resources buffer the impact of job demands on burnout. J Occup Health Psychol. 2005; 10: 170–180. 10.1037/1076-8998.10.2.170 15826226

[24] Schaufeli WB, Bakker AB, Van Rhenen W. How changes in job demands and resources predict burnout, work engagement, and sickness absenteeism. J Organ Behav. 2009; 30: 893–917. 10.1002/job.595

[25] Bakker AB, Demerouti E, Verbeke W. Using the Job Demands-Resources model to predict burnout and performance. Hum Resour Manage. 2004; 43: 83–104. 10.1002/hrm.20004

[26] Bakker AB, Van Veldhoven MJPM, Xanthopoulou D. Beyond the Demand-Control model: Thriving on high job demands and resources. J Person Psychol. 2010; 9: 3–16. 10.1027/1866-5888

[27] Bakker AB, Demerouti E. Towards a model of work engagement. Career Dev Int. 2008; 13: 209–223. 10.1108/13620430810870476

[28] Xanthopoulou D, Bakker AB, Demerouti E, Schaufeli WB. The role of personal resources in the job demands–resources model. Int J Stress Manag. 2007; 14(2): 121–141. 10.1037/1072-5245.14.2.121

[29] Schaufeli WB, Salanova M, González–Romá V, Bakker AB. The measurement of engagement and burnout: A two sample confirmatory factor analytic approach. J Happiness Stud. 2002; 3: 71–92. http://hdl.handle.net/10.1023/A:1015630930326

[30] Leiter MP, Bakker AB. Work engagement: State of the art In: Bakker AB, Leiter MP, editors. Work engagement: A handbook of essential theory and research. New York: Psychology Press; 2010.

[31] Maslach C, Schaufeli WB, Leiter MP. Job Burnout. Annu Rev Psychol. 2001; 52(1): 397–422. 10.1146/annurev.psych.52.1.397 11148311

[32] Halbesleben JRB. A meta-analysis of work engagement: Relationships with burnout, demands, resources, and consequences In: Bakker AB, Leiter MP, editors. Work Engagement: A Handbook of Essential Theory and Research. New York: Psychology Press; 2010 pp. 102–117.

[33] Bakker AB, Demerouti E. Job Demands-Resources theory: Taking stock and looking forward. J Occup Health Psychol. 2017; 22: 273–285. 10.1037/ocp0000056 27732008

[34] Bass BM. Two decades of research and development in transformational leadership. EJWOP. 1999; 8(1): 9–32. 10.1080/135943299398410

[35] Halbesleben JRB, Whitman MV, Crawford WS. A dialectical theory of the decision to go to work: Bringing together absenteeism and presenteeism. Hum Resour Manage R. 2014; 24(2): 177–192. 10.1016/j.hrmr.2013.09.001

[36] Uhl–Bien M. Relational Leadership Theory: Exploring the social processes of leadership and organizing. Leadersh Q. 2006; 17(6): 654–676. 10.1016/j.leaqua.2006.10.007

[37] Mauno S, Mäkikangas A, Kinnunen U. A longitudinal person-centred approach to the job demands-control model. EJWOP. 2016; 25(6): 914–927. 10.1080/1359432X.2016.1187135

[38] Breaugh JA. The work autonomy scales: Additional validity evidence. Hum Relat. 1989; 42(11): 1033–1056. 10.1177/001872678904201105

[39] Roczniewska MA, Puchalska–Kamińska M. Are managers also ‘crafting leaders’? The link between organizational rank, autonomy, and job crafting. Pol Psychol Bull. 2017; 48(2): 198–211. https://doi.org/2017-39804-007

[40] Grant AM, Parker SK. Redesigning work design theories: The rise of relational and proactive perspectives. Acad Manag Ann. 2009; 3(1): 317–375. 10.1080/19416520903047327

[41] Petit WE, Knee CR, Rodriguez LM. Self-determination theory and intimate partner violence: An APIM model of need fulfillment and IPV. Motiv Sci. 2017; 3(2): 119–132. 10.1037/mot0000054

[42] Uhl–Bien M, Pillai R. The romance of leadership and the social construction of followership In: Shamir B, Pillai R, Bligh MC, Uhl–Bien M, editors. Follower-Centered Perspectives on Leadership. Greenwich, CT: Information Age Publishing; 2007 pp. 187–209.

[43] Chaleff I. The Courageous Follower: Standing Up To & For Our Leaders (3rd ed). San Francisco, CA: Berrett-Koehler; 2009.

[44] Hochwarter W, Byrne ZS. LMX and job tension: Linear and non-linear effects and affectivity. J Bus Psychol. 2005; 19(4): 505–520. 10.1007/s10869-005-4522-6

[45] Blau P. Exchange and Power in Social Life. New York: Wiley & Sons; 1964.

[46] Emerson RM. Social exchange theory. Annu Rev Sociol. 1976; 2(1): 335–362. 10.1146/annurev.so.02.080176.002003

[47] Gouldner AW. The norm of reciprocity: A preliminary statement. Am Sociol Rev. 1960; 25: 161–178. 10.2307/2092623

[48] Hinkin TR, Schriesheim CA. An examination of “nonleadership”: From laissez–faire leadership to leader reward omission and punishment omission. J Appl Psychol. 2008; 93(6): 1234–1248. 10.1037/a0012875 19025245

[49] Skogstad A, Hetland J, Glasø L, Einarsen S. Is avoidant leadership a root cause of subordinate stress? Longitudinal relationships between laissez–faire leadership and role ambiguity. Work & Stress. 2014; 28(4): 323–341. 10.1080/02678373.2014.957362

[50] Skogstad A, Einarsen S, Torsheim T, Aasland MS, Hetland H. The destructiveness of laissez–faire leadership behavior. J Occup Health Psychol. 2007; 12(1): 80–92. 10.1037/1076-8998.12.1.80 17257068

[51] Schaubroeck JM, Shen Y, Chong S. A dual-stage moderated mediation model linking authoritarian leadership to follower outcomes. J Appl Psychol. 2017; 102(2): 203–214. 10.1037/apl0000165 27786498

[52] Schyns B, Schilling J. How bad are the effects of bad leaders? A meta-analysis of destructive leadership and its outcomes. Leadersh Q. 2013; 24: 138–158. 10.1016/j.leaqua.2012.09.001

[53] Arnold JA, Arad S, Rhoades JA, Drasgow F. The Empowering Leadership Questionnaire: the construction and validation of a new scale for measuring leader behaviors. J Organ Behav. 2000; 21(3): 249–269. 10.1002/(SICI)1099-1379(200005)21:3<249::AID-JOB10>3.0.CO;2-#

[54] World Medical Association. World Medical Association Declaration of Helsinki. Ethical principles for medical research involving human subjects. Bull World Health Organ. 2001; 79(4): 373–374. 11357217 PMC2566407

[55] Bedeian AG, Hunt JG. Academic amnesia and vestigial assumptions of our forefathers. Leadersh Q. 2006; 17(2): 190–205. 10.1016/j.leaqua.2005.12.006

[56] Carsten MK, Uhl–Bien M, West BJ, Patera JL, McGregor R. Exploring social constructions of followership: A qualitative study. Leadersh Q. 2010; 21: 543–562. 10.1016/j.leaqua.2010.03.015

[57] van Veldhoven MJPM, Prins J, van der Laken PA, Dijkstra L. Manual QEEW2.0: 42 Short Scales for Survey Research on Work, Well–Being and Performance. Amsterdam: SKB; 2015.

[58] Schaufeli WB, Bakker AB, Salanova M. The measurement of work engagement with a short questionnaire. Educ Psychol Meas. 2006; 66: 701–716. 10.1177/0013164405282471

[59] Demerouti E, Mostert K, Bakker AB. Burnout and work engagement: A thorough investigation of the independency of both constructs. J Occup Health Psychol. 2010; 15(3): 209–222. 10.1037/a0019408 20604629

[60] Nunnally JC. Psychometric Theory. 2nd ed New York: McGraw–Hill; 1978.

[61] Kenny DA, Kashy D, Bolger N. Data analysis in social psychology In: Gilbert D, Fiske S, Lindzey G, editors. Handbook of Social Psychology. New York: McGraw–Hill; 1998 pp. 233–265.

[62] Chung ML, Moser DK, Lennie TA, Rayens MK. The effects of depressive symptoms and anxiety on quality of life in patients with heart failure and their spouses: Testing dyadic dynamics using Actor–Partner Interdependence Model. J Psychosom Res. 2009; 67(1): 29–35. 10.1016/j.jpsychores.2009.01.009 19539816 PMC2732117

[63] Stas L, Kenny DA, Mayer A, Loeys T. Giving dyadic data analysis away: A user-friendly App for Actor-Partner Interdependence Models. *Pers Relationships*. 2018; 25(1): 103–119. 10.1111/pere.12230

[64] Fitzpatrick J, Gareau A, Lafontaine MF, & Gaudreau P. How to use the Actor-Partner Interdependence Model (APIM) to estimate different dyadic patterns in Mplus: A step-by-step tutorial. *The Quantitative Methods for Psychology*. 2016; 12(1), 74–86. 10.20982/tqmp.12.1.p074

[65] Leiter MP, Maslach C. The impact of interpersonal environment on burnout and organizational commitment. J Organ Behav. 1988; 9(4): 297–308. 10.1002/job.4030090402

[66] Stenmark CK, Mumford MD. Situational impacts on leader ethical decision-making. Leadersh Q. 2011; 22(5): 942–955. 10.1016/j.leaqua.2011.07.013

[67] Schaufeli WB, Taris TW. A critical review of the Job Demands-Resources Model: Implications for improving work and health In: Bauer G, Hämmig O, editors. Bridging Occupational, Organizational and Public Health. Dordrecht: Springer; 2014 pp. 43–68.

[68] Bakker AB. Strategic and proactive approaches to work engagement. Organ Dyn. 2017; 46: 67–75. 10.1016/j.orgdyn.2017.04.002

[69] Huynh JY, Xanthopoulou D, Winefield AH. The job demands-resources model in emergency service volunteers: Examining the mediating roles of exhaustion, work engagement and organizational connectedness. Work & Stress. 2014; 28(3): 305–322. 10.1080/02678373.2014.936922

[70] Warr P. The measurement of well-being and other aspects of mental health. J Occup Psychol. 1990; 63: 193–210. 10.1111/j.2044-8325.1990.tb00521.x

[71] Neufeld DJ, Wan Z, Fang Y. Remote leadership, communication effectiveness and leader performance. Group Decision and Negotiation. 2010; 19(3): 227–246. 10.1007/s10726-008-9142-x

[72] Perdue CW, Dovidio JF, Gutman MB, Tyler RB. Us and them: Social categorization and the process of intergroup bias. J Pers Soc Psychol. 1990; 59: 475–486. 10.1037/0022-3514.59.3.475

[73] Thody A. Followership in educational organizations: A pilot mapping of the territory. Leadersh Policy Sch. 2003; 2: 141–156. 10.1076/lpos.2.2.141.15542

[74] Meindl JR, Ehrlich SB, Dukerich JM. The romance of leadership. Adm Sci Q. 1985; 30(1): 78–102. 10.2307/2392813

[75] Bakker AB, Xanthopoulou D. The crossover of daily work engagement: Test of an actor–partner interdependence model. J Appl Psychol. 2009; 94(6): 1562–1571. 10.1037/a0017525 19916663

[76] Liden RC, Wayne SJ, Stilwell D. A longitudinal study on the early development of leader–member exchanges. J Appl Psychol. 1993; 78(4): 662–674. 10.1037/0021-9010.78.4.662

[77] Breaugh JA. The measurement of work autonomy. Hum Relat. 1985; 38(6): 551–570. 10.1177/001872678503800604

